# Study on Mandrel Forging and Necking Process of a Hollow Shaft with an Inner Stepped Hole

**DOI:** 10.3390/ma15155431

**Published:** 2022-08-07

**Authors:** Xiqing Ge, Chensheng Tian, Yupeng Lu, Guangchun Wang

**Affiliations:** Key Laboratory for Liquid-Solid Structural Evolution and Processing of Materials (Ministry of Education), Shandong University, Jinan 250061, China

**Keywords:** mandrel forging and necking process, preforming design method, hollow shaft with inner stepped hole, large forgings, free forging

## Abstract

An advanced process of mandrel forging and necking (MFN) was proposed for a hollow shaft with an inner stepped hole. The conventional mandrel forging process with an equal-diameter mandrel was used to form the outer stepped preform, and then the preform was formed into the hollow shaft with an inner stepped hole using the MFN process. A numerical simulation model was established to study the effect of the pressing reduction and the rotation angle on the MFN process. A preforming design method based on the isometric radius difference was given according to the principle of the equal volume, and the parameter relationships between the outer and inner stepped shapes were clarified. The experimental deformation laws of the MFN process were consistent with those obtained by the simulation. The MFN process and its preforming design method provide a new free forging approach for large hollow forgings with inner stepped holes.

## 1. Introduction

Large forgings are essential components of the large and heavy equipment. With the development of electric power, the shipping industry, and nuclear power, the production costs and the quality requirements of large forgings are more stringent [1,2,3]. Large, streamlined forgings with fine microstructures are formed by forging so that the serviceability of the equipment can be ensured, and production time can be saved by the avoidance of reprocessing, welding, and assembly [4,5]. A hollow shaft is a large hollow forging with a hole through it, which is widely used in the fields of hydropower and rail transit [6,7].

Mandrel forging and saddle forging are widely used in free forging for through-hole parts [8,9,10,11]. Jha et al. [12] investigated the mandrel forging process for heavy-duty steel rings. The forming quality was effectively improved by optimizing the preforming structure. Defects were eliminated during the production process, and the microstructure of the forgings was improved. Wang et al. [13] compared the effect of mandrel forging with that of the hot ring rolling process on the deformation laws of cylindrical parts. The results showed that the forgings formed by the hot ring rolling process had better forming quality. Wang et al. [14] analyzed the mandrel reaming process of large cylinders. It proved that the parameters of the forming process were crucial for the quality of the final forgings. Wu and Hu et al. [15,16] studied the mandrel forging process of forming large hollow shafts. Numerical simulations and experiments were conducted to determine the optimal process for reducing forging anisotropy and ensuring forming quality.

**Figure 1:**
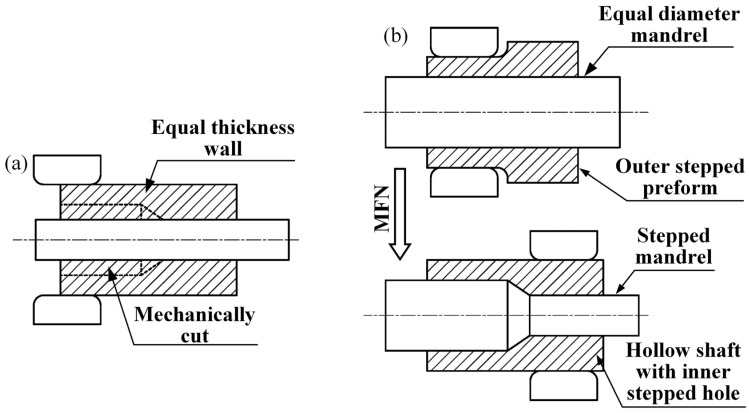
Different processes: (**a**) conventional process; (**b**) mandrel forging and necking (MFN) process.

The mentioned studies were focused on the forming process and property control of large forgings with an equal inner diameter of the through hole. For hollow shafts with an inner stepped hole, the conventional process uses the mandrel to form a hollow shaft with a small inner diameter and a wall of equal thickness, as shown in Figure 1a. Then, the inner hole of the thin-walled zone was mechanically cut to the target size. The conventional process required a large amount of material to be removed in this zone [17]. Wasted material and machining time, as well as a large number of forming lines at the inner stepped hole were cut off [18]. The conventional process severely diminished the strength and toughness of the local zone at the inner stepped hole and the integral hollow shaft because the cutting process cut off the forming lines and weakened the properties [19].

In this study, an advanced mandrel forging and necking (MFN) process was proposed for a hollow shaft with an inner stepped hole, as shown in Figure 1b. The outer stepped preform was first formed by using an equal-diameter mandrel with the same size as the large inner stepped hole, and then a stepped mandrel with the same size as the inner stepped hole was used to form the small hole end with the MFN process. The characteristics of the deformation distribution and law in the deformation zone of the MFN process were investigated. The preforming design method for the outer steeped preform was proposed based on the principle of the equal volume and the isometric radius difference. The proposed MFN process and the preforming design method were confirmed to be capable of controlling the quality problems, such as the inner hole distortion and surface-folding defects, under the numerical simulation and experiment. These provide guidance for the production of large hollow shafts with inner stepped holes.

## 2. Preforming Design Method

**Figure 2:**
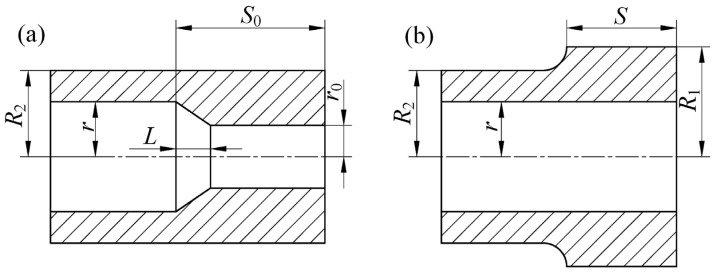
Schematic diagram of preforming design method: (**a**) target forging; (**b**) preform.

The target forging with a beveled transition at the inner stepped hole is shown in Figure 2a. For this target forging, the outer radius of the target forging is *R*_2_, the large radius of the inner stepped hole is *r*, the small radius is *r*_0_, the target length of the MFN section is *S*_0_, and *L* is the length of the inner hole transition zone. Figure 2b shows the outer stepped preform formed by conventional mandrel forging with an equal-diameter mandrel. The large outer radius is *R*_1_, and the preformed MFN length is *S*. Axial material loss and material transfer between thick and thin wall zones are neglected during the MFN process. The volumes of before and after the MFN process are expressed in Equations (1) and (2), respectively.

(1) $$V_{0}=\pi S_{0}(R_{2}^{2}{-r}_{0}^{2})+\pi r_{0}^{2}L-\frac{1}{3}\pi (r_{0}^{2}+r^{2}+rr_{0})L$$

(2) $$V=\pi (R_{1}^{2}-r^{2})S$$

where *V*_0_ is the MFN volume of the target forging and *V* is the preformed MFN volume.

According to the principle of the equal volume in metal forging, the relationship between *S* and *R*_1_ of the preform is established by *V*_0_ equals *V*.

(3) $$S=\frac{S_{0}(R_{2}^{2}{-r}_{0}^{2})+r_{0}^{2}L-\frac{1}{3}(r_{0}^{2}+r^{2}+rr_{0})L}{R_{1}^{2}-r^{2}}$$

The preform shape in Figure 2b can be obtained in accordance with Equation (3) as long as the associated change law of *R*_1_ and *S* is present in the MFN process.

**Figure 3:**
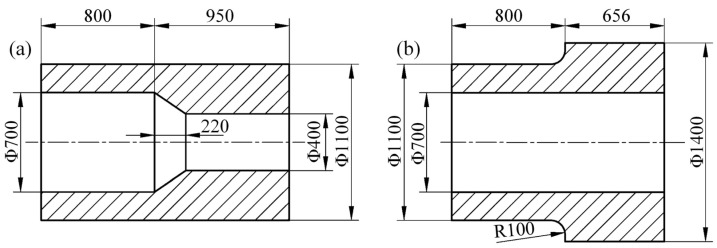
Sizes of target forging and preform (unit: mm): (**a**) target forging; (**b**) K2 preform.

In accordance with the size of target forging, the necking section was shrunk from Φ700 mm to Φ400 mm as shown in Figure 3a. The large inner diameter zone (Φ700 × 800 mm) of the target forging was formed directly by mandrel forging with the equal-diameter mandrel. To investigate the effect of *R*_1_ and *S* on forming quality, and to obtain the preforming design method, the different preform shapes are shown in Table 1. The value of *R*_1_ and *S* in the preform should be defined by Equations (1)–(3) when the target forging size is constant. ∆*S* is the difference between the target length *S*_0_ and the preformed length *S* of the MFN process. The difference ∆*r* = 150 mm in the radius of the target forging was taken as a reference to design the different preformed outer stepped radius difference, ∆*R*. The K2 scheme was used for the subsequent simulations and experiments aiming to initially determine the deformation law and the effect of different parameters on the MFN process. The K2 preform is shown in Figure 3b.

## 3. Numerical Simulation Model and Experimental Procedure

### 3.1. Establishment of Numerical Simulation Model

**Figure 4:**
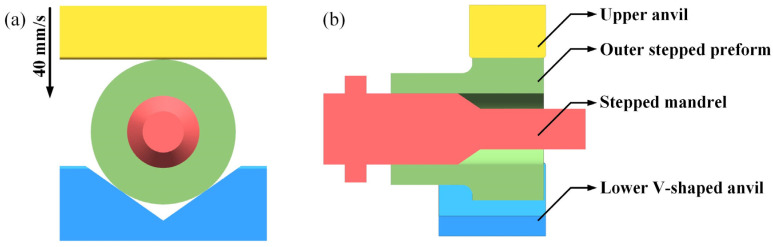
Numerical model for the MFN process: (**a**) front view; (**b**) right-side view.

Transvalor Forge Nxt1.1 software [20] was applied to analyze the effective strain distribution in the deformation zone and the material deformation law during the MFN process [8,21]. The numerical simulation model is shown in Figure 4, which included an upper anvil, a stepped mandrel, a lower V-shaped anvil, and an outer stepped preform.

**Figure 5:**
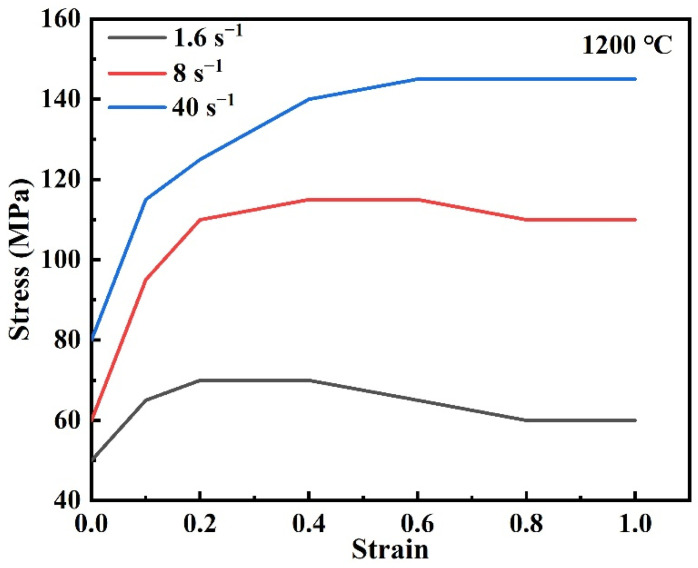
Flow stress of 42CrMo4 at 1200 °C obtained from the software.

The billet was defined as plastic, and the mandrel and anvils were set as rigid without deformation. The surface nodes of the billet were sliding along the surface of the anvils with a shear friction type. The large forgings were formed without lubrication during hot deformation. Therefore, the Forge software provided a friction factor of 0.4 using the friction condition of “no lube”. The meshing method was the automatic re-meshing method, and the mesh number of the billet was 150,000. The material was subjected to a large plastic deformation in the MFN process, and the elastic deformation was negligible. The viscoplastic constitutive model was used as shown in Equation (4). The flow stress is related to the strain, strain rate, and temperature. Figure 5 shows the flow stress of materials at 1200 °C obtained from the software.

(4) $$\bar{\sigma }=\bar{\sigma }(\bar{\varepsilon },\;\overset{\cdot }{\bar{\varepsilon }},\;T)$$

where $\bar{\sigma }$ is flow stress, $\bar{\varepsilon }$ is strain, $\overset{\cdot }{\bar{\varepsilon }}$ is strain rate, and *T* is temperature.

A multi-passes press was used to form a hollow shaft with an inner stepped hole. When the width of the upper anvil was less than the length of the MFN section after one loop, it was necessary to move the upper anvil along the axial direction to stagger the previous deformation zone for the next position and loop of the MFN process as the conventional mandrel forging. The preheating temperatures of the billet and anvils were 1200 °C and 300 °C, respectively. The heat transfer coefficients between the billet with the atmosphere and anvils were 10 W/(m^2^·°C) and 20,000 W/(m^2^·°C), respectively. The upper anvil was pressed at 40 mm/s with an interval of 5 s. The angle of the V-shaped anvil was 110° [22]. The detailed parameters of the numerical simulation are shown in Table 2.

### 3.2. Experimental Procedure

It was infeasible to conduct a hot die forging experiment with equal ratios and conditions because the production equipment required for large forgings is huge and complicated to operate. The recrystallization temperature of pure lead is approximately −17 °C [23]. The deformation flow behavior at room temperature is similar to that of carbon steels above recrystallization temperatures, such as that of 42CrMo4. In addition, the friction factor and the heat transfer coefficient of lead at room temperature are similar to 42CrMo4 steels above the recrystallization temperature [8]. The lead shrinkage ratio experiments with a shrinkage ratio of 25:1 were conducted on a 10T universal testing machine (SANS CMT5105).

## 4. Results and Discussion

### 4.1. Single-Step Pressing Deformation Analysis

**Figure 6:**
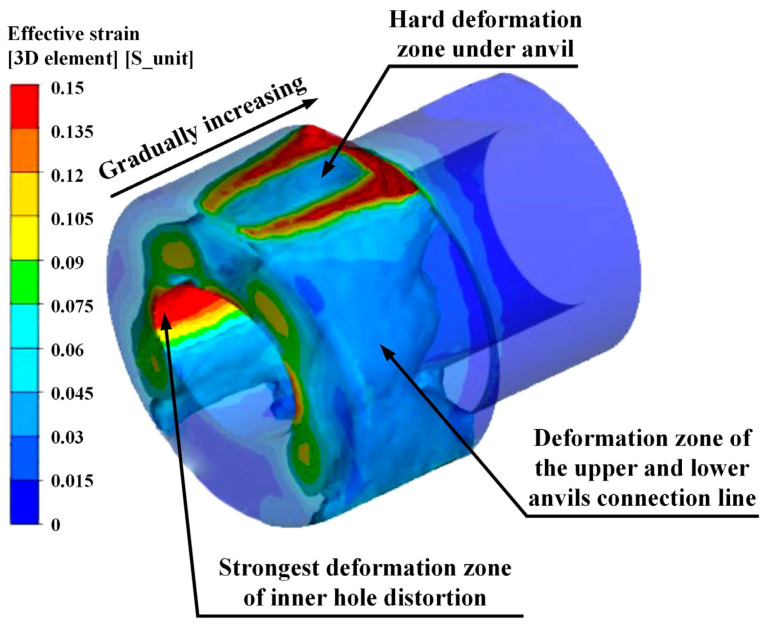
Effective strain distribution of the three-dimensional integral.

The preform and the simulation model are shown in Figure 3b and Figure 4. The deformation zone in the MFN process was mainly concentrated in the necking section. Single-step pressing was simulated with a pressing reduction of 40% in the wall thickness. The effective strain distribution of the three-dimensional integral is illustrated in Figure 6. The transparent areas in the figure showed the no-deformation zone, and the zone with an effective strain distribution greater than 0.06 was shown as opaque. The results demonstrated that the effective strain distribution of axial deformation was unevenly distributed. From the free end along the axis direction, the effective strain distribution of the hard-deformation zone between the upper anvil and preform gradually increased inward, and it distributed symmetrically from left to right. The effective strain distribution of the upper and lower anvils connection line decreased progressively from the free end inward. The effective strain distribution of the strongest deformation zone was the largest due to the hole distortion, which was more uniformly distributed in the axial direction.

**Figure 7:**
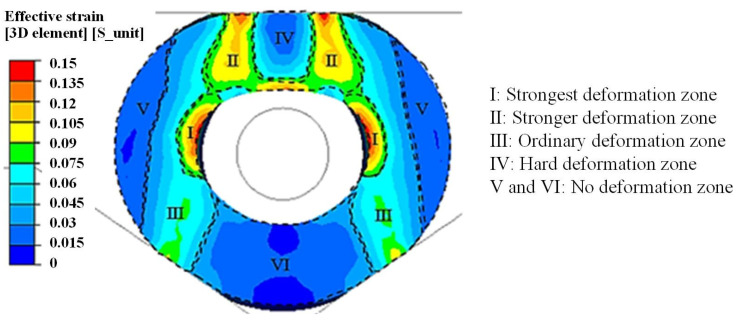
Effective strain distribution of single-step pressing on the cross-section.

The inner hole was not restrained by the stepped mandrel, which was different from conventional mandrel forging. The integral shape of the inner hole was an up-down asymmetrical ellipse. The effective strain distribution of the cross-section is shown in Figure 7. The material in the upper sidewall zone of the inner hole was transferred downward. The inner hole shape was flattened, and the curvature was remarkably decreased. The curvature of the inner hole zone corresponding to the V-shaped anvil had a slight modification, and the hole size was slightly reduced. The hole diameter was elongated as the material flowed radially outward on both sides of it. 

The cross-section was divided into six zones, I–VI, according to the effective strain distribution of the deformation zones, as shown in Figure 7. The inner hole was pressed by the upper and lower anvils. The strongest deformation zone I, with a symmetrical crescent shape, was formed. As shown in zone II, the resistance to deformation of material was small, and the deformation was strong at the edge of the interface between the upper anvil and the preform. The upper and lower anvils were attached to the contact point of the preform in zone III. Zone IV was the hard-deformation zone below the upper anvil. There was no deformation in zones V and VI.

**Figure 8:**
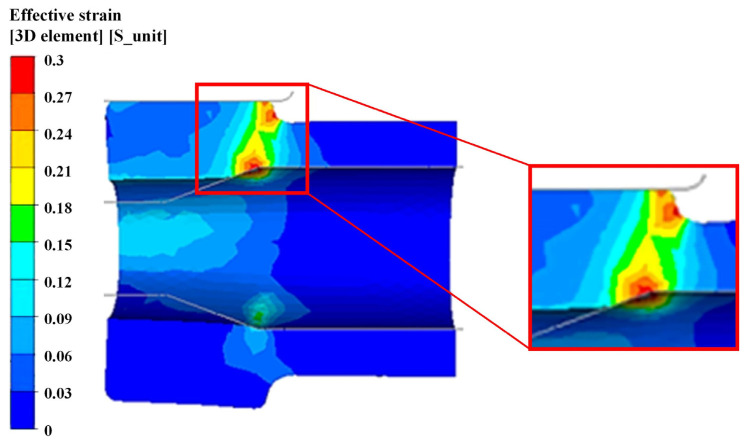
Effective strain distribution of single-step pressing on the longitudinal section.

The effective strain distribution of single-step pressing on the longitudinal section was shown in Figure 8. The maximum strain occurred at the mandrel transition zone during the MFN process. The material was subjected to similar shear stress with the maximum deformation. It was clear from the partial enlargement in Figure 8 that the deformation of the upper zone was significantly larger than that of the lower zone.

### 4.2. The Effect of the Pressing Reduction

The pressing reduction was the most critical parameter in the MFN process. The choice of the pressing reduction directly affected the deformation zone and the hole distortion. The preform and the simulation model are shown in Figure 3b and Figure 4. The different pressing reductions ∆*h* of 40 mm, 80 mm, 120 mm, and 140 mm (11%, 22%, 34%, and 40%) were selected for the MFN process. Four schemes of pressing reduction were designed to investigate the effective strain distribution of the deformation zones on the cross-section.

**Figure 9:**
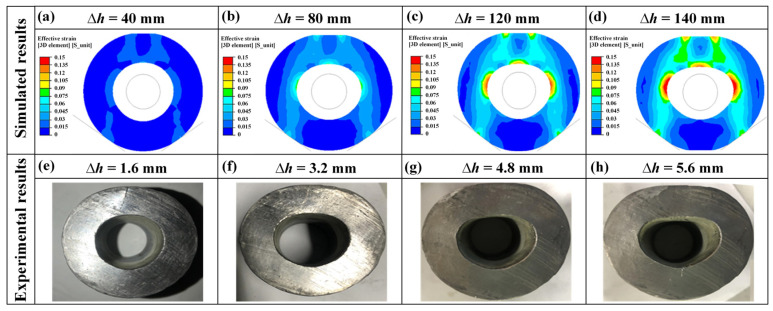
Deformation under different pressing reductions: (**a**–**d**) simulated results; (**e**–**h**) experimental results with a shrinkage ratio of 25:1.

As shown in Figure 9a–d, the deformation zones gradually enlarged with the increase in the pressing reductions. Meanwhile, the simulated pressing reduction was used in the 25:1 shrinkage ratio experiment. The pressing reductions ∆*h* were set to 1.6 mm, 3.2 mm, 4.8 mm, and 5.6 mm. The results of the single-step pressing experiment are shown in Figure 9e–h, and it could be seen that the inner hole distortion in the experiment was similar to the simulation.

**Figure 10:**
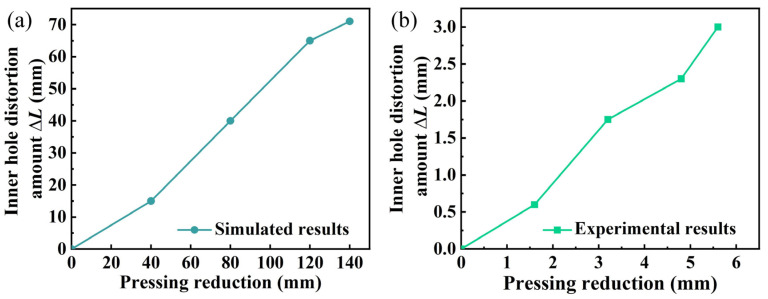
Distortion trend of the inner hole with different pressing reductions: (**a**) simulated results; (**b**) experimental results with a shrinkage ratio of 25:1.

∆*L* was defined as the inner hole distortion, which was the difference between the maximum size of the former and the latter inner hole diameters. As shown in Figure 10a, ∆*L* exhibited a linear growth trend with the increase in the pressing reduction. Figure 10b illustrated that ∆*L* of the shrinkage ratio experiments also showed a linear growth trend.

As shown in Figure 9d,h, when the pressing reduction was large, the distortion of the inner hole was severe and it would appear that the pressing reduction was uneven. The preform was not constrained by the mandrel, resulting in the inferior forming quality. The roundness was substandard, and folding defects occurred. Increasing the pressing reduction would improve the productivity; however, excessive pressing reduction would result in the poor forming quality of the inner hole, such as folding defects. As shown in Figure 9b, the deformation zones obtained in ∆*h* of 120 mm and 140 mm were essentially the same as 80 mm. The strain-distribution zones were basically deformed, and the inner hole distortion ∆*L* was about 35 mm. As a result, it was recommended that a smaller pressing reduction, with about 20% of the wall thickness, was beneficial to forming strain-distribution zones, and would be conducive for subsequent pressing corrections with the purpose of ensuring the forming quality.

### 4.3. The Effect of the Rotation Angle

The articulation of two deformations was determined by the rotation angle of the preform, and it also determined the uniformity of the deformation distribution and the roundness of the inner hole. Three rotation angle schemes were based on a pressing reduction of 20%, as shown in Table 3.

**Figure 11:**
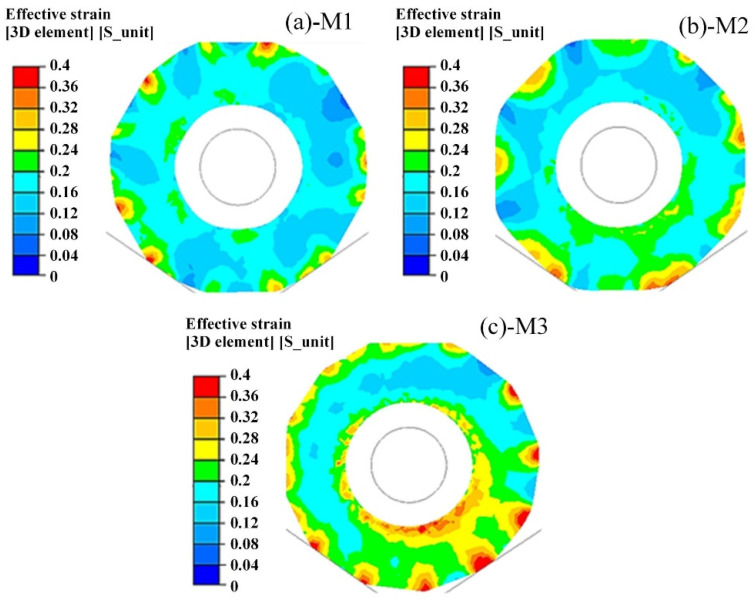
Effective strain distribution under different rotation angle methods: (**a**) M1; (**b**) M2; (**c**) M3.

The outer (inner) diameter difference was indicated by the roundness of the horizontal deformation. As shown in Figure 11a, there was a hexagonal inner hole of the preform with sharp angles in the outer profile. The M2 scheme, with increased number of pressing reduction and reduced rotation angle, was compared with the outer (inner) diameter difference in Table 3. Comparing M2 with M1 and M3, it could be inferred that M2 significantly enhanced the roundness of the inner and outer holes. The effective strain distribution of different rotation angle methods on the cross-section in Table 3 was shown in Figure 11. All three rotation angle schemes presented uneven deformation. The M1 scheme had the majority of small-strain areas (less than 0.2) in the cross-section, as shown in Figure 11a. It appeared that the overlap of the deformation zone between the two passes was insufficient, and the forming effect was inadequate. The roundness of M2 and M3 was similar, however, the larger strain areas of the latter were more than that of the former in terms of the effective strain distribution, as shown in Figure 11b,c. The M3 scheme enabled better integration of the hard-deformation zone under the anvil, and the effective strain in the surface reached above 0.2. In summary, the smaller the rotation angle, the better the inner hole forming quality. The M3 scheme was preferred in the case of high-quality requirements, and the M2 scheme could be adopted when the inner hole quality requirements were average.

**Figure 12:**
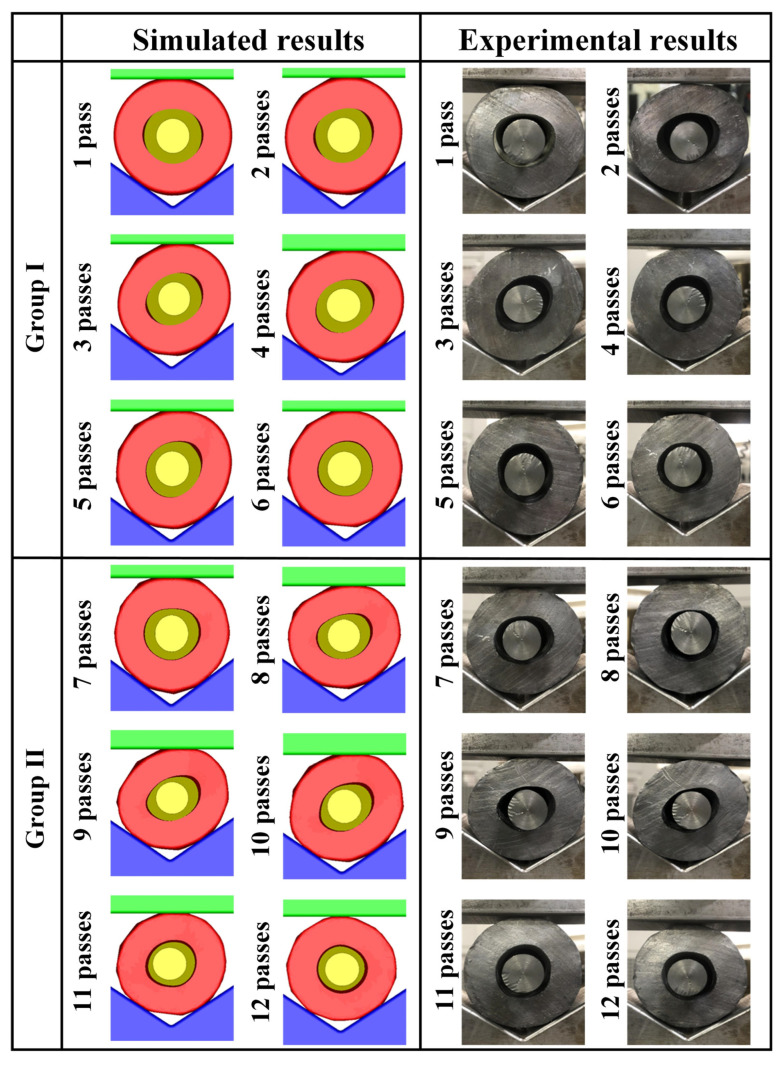
The MFN process of M3 scheme (the experiments with shrinkage ratio of 25:1).

**Figure 13:**
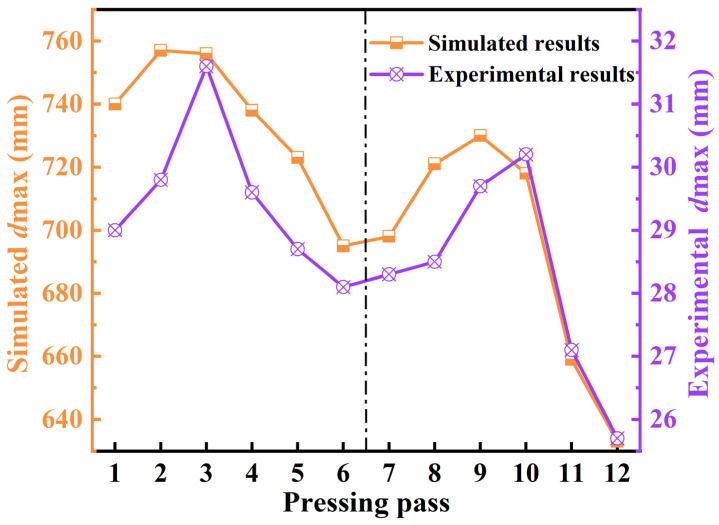
Inner hole distortion of M3 scheme (the experiments with shrinkage ratio of 25:1).

The M3 scheme was used for shrinkage ratio experiment to verify the accuracy of the simulation. The results are shown in Figure 12. The M3 scheme required a total of 12 passes of one loop, which was divided into two groups. Passes one–six were those of Group Ⅰ and the rest were those of Group Ⅱ. As shown in Figure 12, there were similar laws of the inner hole distortion for two groups. The inner hole distortion gradually increased and then corrected during one loop. Figure 13 shows the one-loop variation of the maximum inner diameter, *d*_max_. The simulated results were similar to the experimental results. *d*_max_ peaked during the third and ninth passes. The pressing reduction would fluctuate with the deformation in the actual production. To enhance the roundness of the inner hole and eliminate folding defects, the pressing reduction could be increased at the third and ninth passes according to the actual inner hole distortion.

### 4.4. The Parameters of the Preform

It was concluded from the production experiment that a preform with larger *R*_1_ and smaller *S* was more favorable to the MFN process. Excessive *R*_1_ would lead to excessive size difference in the transition zone, which would be prone to fold defects. Meanwhile, it increased the pressing reduction and the loop, which affected the production rhythm and reduced productivity. The simulation schemes for different preform shapes are shown in Table 1. The rotation angle of 12 × 30° with the pressing reduction of 20% was used. 

**Figure 14:**
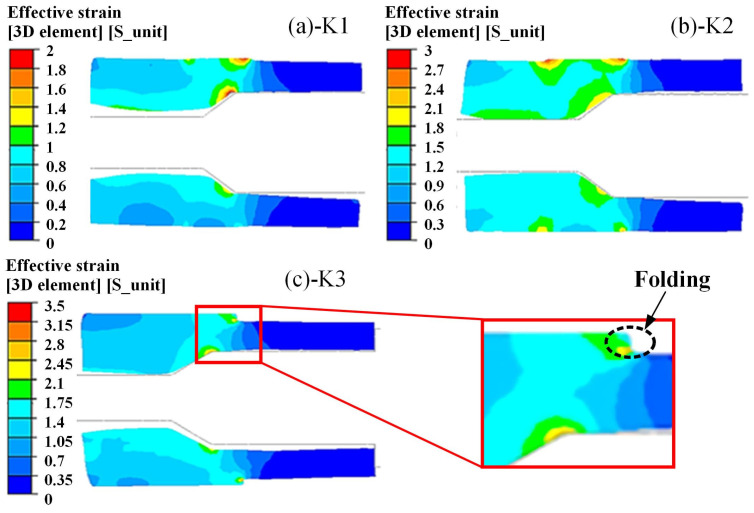
Results of different preforming design schemes: (**a**) K1; (**b**) K2; (**c**) K3.

As shown in Table 1, The K2 scheme was the isometric radius difference of the preforming design method. When *R*_1_ was R700 mm, the difference of the MFN radius was consistent with the difference of the preformed outer stepped radius (*r* − *r*_0_ = *R*_1_ − *R*_2_). The results of the different preforming design schemes are shown in Figure 14. The smaller radius *R*_1_ resulted in the thinnest wall thickness by using the (a)-K1 scheme. The inner hole was not necked to the target size after pressing to the required outer diameter. The MFN section was properly shaped under the (b)-K2 scheme. There was a portion of the material that did not fit the mandrel, but it was below the machining allowance. The folding appeared at the stepped transition after two loops using the (c)-K3 scheme.

In summary, it was demonstrated that the simulated forming quality and sizes were ideal using the design method of the isometric radius difference. The preformed outer stepped radius difference was the same as the stepped hole radius difference of the target forging.

### 4.5. The Shrinkage Ratio Experiment

**Figure 15:**
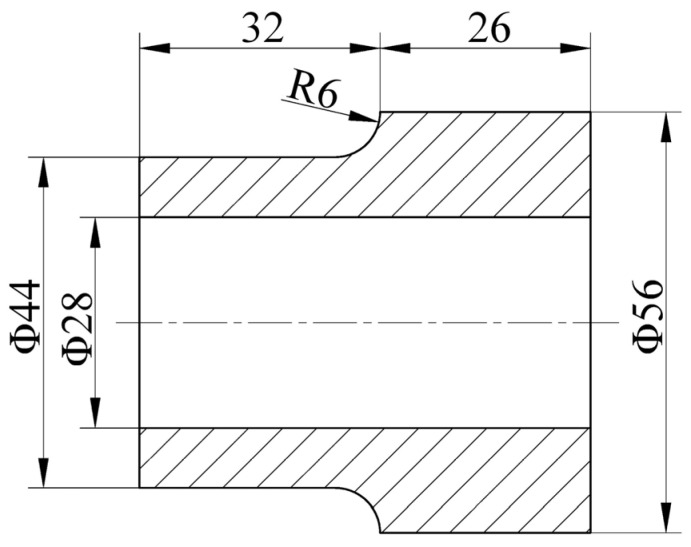
Preform of shrinkage ratio experiment (unit: mm).

The preform shape and sizes for the shrinkage ratio experiment are shown in Figure 15 according to the preforming design method and the isometric radius difference method. The 25:1 shrinkage ratio experiment with four loops was carried out using a pressing reduction of 20% and a rotation angle of 12 × 30°.

**Figure 16:**
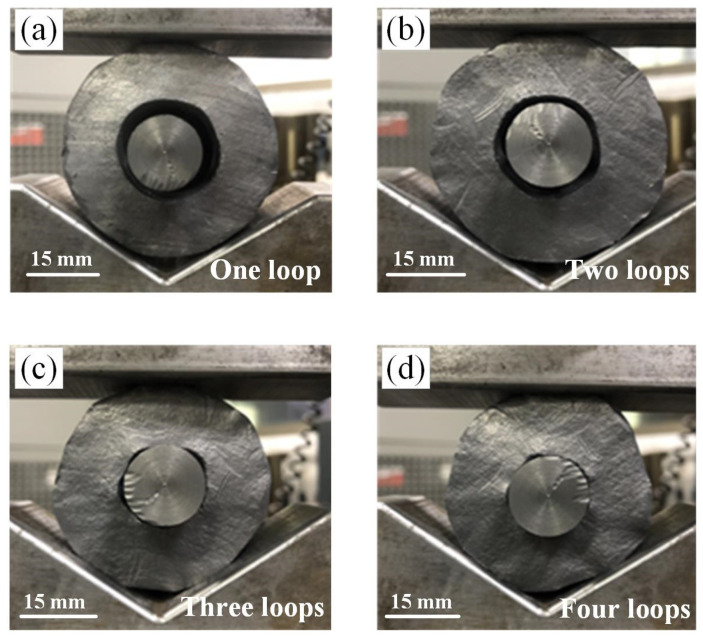
The inner hole variation of the shrinkage ratio experiment: (**a**) one loop; (**b**) two loops; (**c**) three loops; (**d**) four loops.

**Figure 17:**
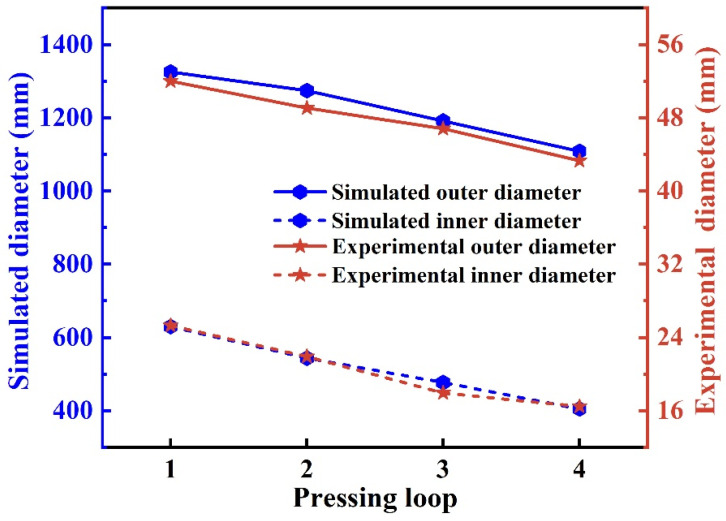
The simulated and experimental variations of inner and outer diameters (the experiments with a shrinkage ratio of 25:1).

The inner hole variation of the shrinkage ratio experiment is shown in Figure 16. The forming of the MFN section was observed, and the simulation results corresponding to the same experimental conditions were measured, as shown in Table 4. The diameter fluctuated because the shrinkage rate was not the same in the longitudinal section of the inner hole. The simulated and experimental variations of the inner and outer minimum diameters are shown in Figure 17.

As shown in Figure 17, the inner and outer diameters of the preform changed linearly with the increase in the pressing loops. It indicated that the change of wall thickness at the deformed section was quite slim in the MFN process. The simulated results were maintained at 347–365 mm, and the shrinkage ratio experimental results were maintained at 13.3–14.4 mm. The difference between the inner and outer diameters (wall thickness of the preform) was basically constant. It further demonstrated the feasibility of the isometric radius difference method.

## 5. Conclusions

This paper studied the forming process of a large hollow shaft with an inner stepped hole. An advanced mandrel forging and necking (MFN) process and its preforming design method were proposed, combined with the numerical simulation and a shrinkage ratio experiment. The results are as follows:

1. An advanced MFN process was proposed for the production of a hollow shaft with an inner stepped hole. The outer stepped hollow shaft was preformed by using a mandrel with the same large hole size, and then the inner stepped hollow shaft was formed by the MFN process using a stepped mandrel with the same size as the inner hole of the target forging.
2. The isometric radius difference of the preforming design method was obtained. The radius difference of the outer stepped preform was identical to that of the hollow shaft with inner stepped hole. The relationship between the size of the preform and the target forging was established.
3. The law of single-step pressing deformation of the MFN process was analyzed. The deformation morphology was divided into six deformation zones. The deformation of each zone was clearly identified to facilitate the subsequent analysis.
4. The parameters of the MFN process were optimized. The pressing reduction was controlled at about 20% of the wall thickness, and the 12 × 30° of rotation angle scheme was utilized. The pressing reduction could be appropriately increased in the third and ninth passes according to the actual inner hole distortion. It guaranteed the quality of the MFN process and eliminated surface-folding defects.

## Figures and Tables

**Table 1:** Different preforming design schemes.

Scheme	*R*_1_/mm	∆*R*/mm	*S*/mm	∆*S*/mm
K1	650	100	804	146
K2	700	150	656	294
K3	750	200	548	402

**Table 2:** Numerical simulation parameters.

Parameters	Value
Mesh number for billet	150,000
Forging temperature	Billet temperature: 1200 °C Anvils temperature: 300 °C
Heat transfer coefficient	Billet and atmosphere: 10 W/(m^2^·°C) Billet and anvils: 20,000 W/(m^2^·°C)
Friction factor	0.4
Pressing velocity	40 mm/s
Pressing interval	5 s

**Table 3:** Different rotation angle methods.

Scheme	Pressing Method	Outer Diameter/mm	Outer Diameter Difference/mm	Inner Diameter/mm	Inner Diameter Difference/mm
M1	6 × 60°	Max: Φ1375 Min: Φ1315	60	Max: Φ685 Min: Φ655	30
M2	8 × 45°	Max: Φ1365 Min: Φ1322	43	Max: Φ680 Min: Φ662	18
M3	12 × 30°	Max: Φ1357 Min: Φ1320	37	Max: Φ675 Min: Φ661	14

**Table 4:** The variation of outer and inner diameters.

	One Loop	Two Loops	Three Loops	Four Loops
Simulated outer diameter/mm	1324.97	1274.04	1191.15	1108.21
Simulated inner diameter/mm	629.44	543.47	476.93	404.61
Experimental outer diameter/mm	51.98	49.06	46.80	43.30
Experimental inner diameter/mm	25.30	21.90	17.92	16.46

## Data Availability

The raw/processed data required to reproduce these findings cannot be shared at this time as the data also form part of an ongoing study.
